# Cytokine Secretion in Macrophages: SNAREs, Rabs, and Membrane Trafficking

**DOI:** 10.3389/fimmu.2014.00538

**Published:** 2014-10-27

**Authors:** Rachael Zoe Murray, Jennifer Lea Stow

**Affiliations:** ^1^Institute of Health and Biomedical Innovation, Queensland University of Technology, Brisbane, QLD, Australia; ^2^Institute for Molecular Bioscience, The University of Queensland, Brisbane, QLD, Australia

**Keywords:** cytokine secretion, macrophages, SNAREs, Rabs, TNF, IL-6, IL-10

## Abstract

Macrophages have the capacity to rapidly secrete a wide range of inflammatory mediators that influence the development and extent of an inflammatory response. Newly synthesized and/or preformed stored cytokines and other inflammatory mediators are released upon stimulation, the timing, and volume of which is highly regulated. To finely tune this process, secretion is regulated at many levels; at the level of transcription and translation and post-translationally at the endoplasmic reticulum (ER), Golgi, and at or near the cell surface. Here, we discuss recent advances in deciphering these cytokine pathways in macrophages, focusing on recent discoveries regarding the cellular machinery and mechanisms implicated in the synthesis, trafficking, and secretion of cytokines. The specific roles of trafficking machinery including chaperones, GTPases, cytoskeletal proteins, and SNARE membrane fusion proteins will be discussed.

## Introduction

Macrophages activated by contact with pathogens or danger signals release cytokines and chemokines as a major component of the innate immune response (1). Inflammatory cytokines recruit other immune cells and orchestrate the actions and fates of the cells secreting them and those in the surrounding milieu. Macrophages are one of the most abundant sources of inflammatory cytokines, which normally act in a protective manner; however, these same cytokines underlie many acute and chronic inflammatory diseases. Increasingly, it emerges that macrophages secrete cytokines inappropriately in an ever broadening array of chronic conditions, from diabetes and obesity to vascular and neurodegenerative diseases (2–4). Many of the newer therapeutics target cytokines directly, their modes of release, or receptor engagement.

Macrophage cytokines are synthesized and released in response to activation of pattern recognition receptors or inflammasomes, and there is an abundant literature documenting the many factors, signaling cascades, and transcriptional machinery that lead to cytokine synthesis (5–7). The subsequent organelles, secretory pathways, and cellular machinery that transport cytokines through the cell are also critical for ensuring timing and site of release for new cytokines. These pathways and their regulators will be the subject of this review. In particular we focus on two large families of trafficking regulators that guide cytokine secretion through multiple intracellular steps, the Rab small GTPases, and the SNAREs (Soluble NSF Attachment Protein Receptors).

## Cytokine Trafficking Pathways

**Figure 1:**
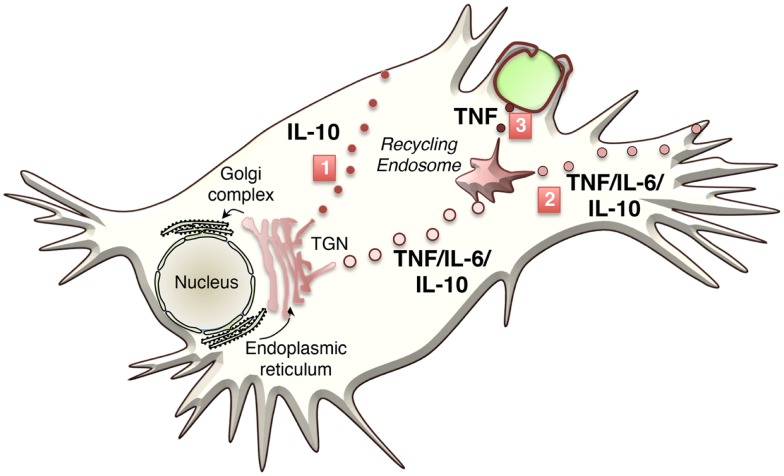
**Classical transport pathways used to secrete macrophage cytokines**. Three major transport pathways for cytokine secretion have been identified to date. The first involves direct transport to the cell surface (IL-10) from the TGN, the second pathways routes cytokines via the recycling endosome and the out to the cell surface (TNF, IL-6, and IL-10), and the 3rd pathway occurs during phagocytosis where cytokine (TNF) is routed from the recycling endosome to the phagocytic cup.

Cytokine secretion pathways are often adapted to suit specific cytokines, their function, and cell type. Many immune cells stockpile cytokines in distinct compartments – namely, secretory granules or lysosome-related organelles (LROs) – that enable rapid release of the cytokines upon cell activation (8, 9). Macrophages, however, lack these granules, and instead cytokines must be synthesized after cell activation and secreted via the constitutive (or continuous) secretory pathway or via non-conventional secretion. The majority of cytokines in macrophages are processed and transported through the constitutive pathway (10–13) (Figure 1). To accommodate the need to transport and release large volumes of cytokines in the first hours after cell activation, the cellular machinery and carriers involved in the constitutive pathway are upregulated (11, 12, 14–16) (Table 1). Much of our knowledge of this pathway comes from work in other cell types, such as epithelial cells, in addition to studies in macrophages themselves. Multiple cell types have in common routing of newly synthesized proteins from the endoplasmic reticulum (ER) to the Golgi complex, sorting in the trans-Golgi network TGN and then for some cargo, transport directly to the cell surface or transit via recycling endosomes (17, 18). Much less is known about the non-classical secretory pathways for cytokine secretion. IL-β and IL-18 are cytokines secreted from the cytoplasm by non-classical pathways, although the exact route of release is contentious and might, in some cases, be cell type specific.

**Table 1:** **Proteins and lipids altered by LPS stimulation**.

Organelle	Protein/lipid	Proposed function	Reference
ER	iRhom2	Promotes TACE exit from the ER	(19)
TGN	PtdChol	Lipid raft/vesicle biogenesis	(20)
TGN	CCTα	Catalyzing PtdChol biosynthesis	(20)
TGN	p230	Sorting at the TGN	(14)
TGN	Rab6	Recruitment/stabilization of p230 on TGN membranes	(15)
TGN	Stx6	Fusion of TGN-derived vesicles with recycling endosome	(12)
TGN	Stx7	Fusion of TGN-derived vesicles with recycling endosome	(12)
TGN	Vti1b	Fusion of TGN-derived vesicles with recycling endosome	(12)
TGN	SCAMP5	Forms a complex with Stx6 and potentially regulates fusion with RE	(21)
RE	VAMP3	Fusion of TGN-derived vesicle with RE and the RE with PM	(11)
RE	Rab11	Fusion of RE with PM	(11)
RE	Rab37	Fusion of RE with PM	(16)
PM	Stx4	Fusion of RE with PM	(22)
PM	SNAP23	Fusion of RE with PM	(22)
PM	SCAMP5	Forms a complex with Stx4/SNAP23 to regulate exocytosis	(21)

*ER, endoplasmic reticulum; TGN, trans-Golgi network; RE, recycling endosome; PM, plasma membrane*.

### Constitutive secretion of cytokines

Cytokines such as IL-2, 3, 6, 10, 12 and TNF contain a signal peptide targeting them to the ER, where once correctly folded, they are packaged into coat protein complex II (COPII) coated vesicles and delivered to the ER–Golgi intermediate compartment (ERGIC) and the *cis* side of the Golgi complex. From here, they are transported through the medial-Golgi to the TGN, a tubular network that emanates from the stacked reticulated ribbon-like structured Golgi cisternae. In addition to the final modifications, proteins might receive in the TGN, this compartment acts as one of the major organizing centers to sort cargo to different post-Golgi carriers and routes (17). This includes the sorting of transmembrane cytokines, such as TNF, and less obvious sorting – but certainly packaging – of soluble cytokines such as IL-6 and IL-10. Other cargo is also segregated at the TGN, including, glycosyl phosphatidyl inositol (GPI)-anchored proteins and lysosomal enzymes with mannose-6-phosphate tags.

Newly synthesized proteins, recycling, and endosomally derived proteins all converge at the TGN as cargo requiring packaging into carriers for post-Golgi transport. Several types of pleomorphic membrane-bound carriers, which can appear as tubules or vesicles and often do not have definable coats, exist within the constitutive secretory pathway. Carrier loading, formation, and transport from the TGN occur in distinct steps. Firstly, cargo must be segregated from resident Golgi proteins and sorted into budding carriers. At exit points on the TGN membrane, a complex array of machinery governs membrane curvature and fission of budding vesicles or tubules, which then use motors to attach to microtubules for transport. Imaging in live macrophages shows that cytokines at the TGN are loaded into tubular or pleomorphic vesicular carriers and the number of carriers forming can be enhanced to accommodate the increased flow of cargo, including cytokines, in activated cells (14, 15).

**Figure 2:**
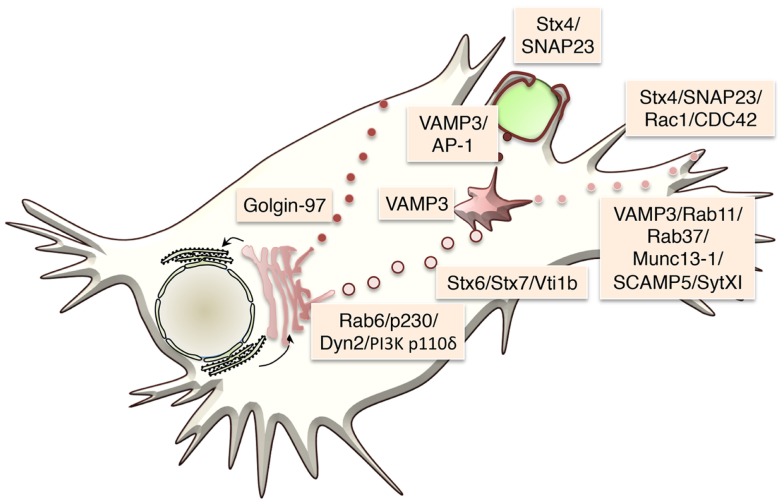
**Trafficking machinery regulating specific cytokine transport steps in macrophages is shown**. The transport machinery that regulates the three major transport steps is shown.

For many cytokines, the TGN sorting mechanisms are unknown or not well defined. In general, the sorting of transmembrane proteins occurs via adaptor recognition of sorting motifs in their cytoplasmic tails or by clustering of cargo proteins in distinct membrane domains such as lipid rafts. ARF and Rab GTPase family members, adaptors, golgins, and lipids, such as phosphoinositides, sphingolipids, and cholesterol, all contribute to cargo sorting and carrier loading at the TGN (17) (Table 2). How soluble cargo, including soluble cytokines such as IL-6 or IL-10, is sorted is still not clear. The transmembrane cytokine TNF is generally sorted into specific carriers, while soluble cytokines often appear in more than one carrier type and exit via more than one pathway. The latter is suggestive of less stringent or perhaps even no effective sorting, of soluble cytokines. While the transmembrane protein TNF is delivered to highly specific sites on the cell surface, the surface delivery sites for release of soluble cytokines may be less regulated. Tubular or vesicular carriers move along microtubule tracks to their destinations. In addition to their cargo, carriers must also be loaded with the necessary machinery for their docking and fusion at the target membrane – steps that frequently involve specific members of the Rab family of small GTPases and of the SNARE family of membrane fusion proteins (Figure 2).

**Table 2:** **Trafficking machinery that regulate cytokine secretion in macrophages**.

Protein	TNF	IL-6	IL-10	Reference
iRhom2	+	NA	NA	(19 )
p230	+	+	+	(14)
Golgin-97	−	−	+	(13)
CCTα	+	+	ND	(20)
PI3K p110δ	+	ND	−	(23)
Dynamin 2	+	ND	ND	(23 )
Stx4	+	ND	+	(22)
Stx6	+	+	+	(12)
Vti1b	+	+	+	(12)
VAMP3	+	+	+	(11)
SNAP23	+	ND	+	(22)
SCAMP5	+	+	ND	(21)
Rab6	+	ND	ND	(15 )
Rab11	+	ND	+	(11)
Rab37	+	ND	ND	(16 )
Munc13-1	+	ND	ND	(16 )
AP-1	+	ND	ND	(24 )
Syt XI	+	+	ND	(25)
PKA	ND	ND	+	(13)
Rac1	+	ND	ND	(26 )
CDC42	+	ND	ND	(26 )

*NA, not applicable; ND, not determined*.

### Membrane fusion and mapping pathways with SNAREs and Rabs

Members of the SNARE family are in general ubiquitously expressed, with the exception of two neuronal specific SNAREs, Stx1, and SNAP25, and the immune specific SNARE Stx11 (27, 28). Each SNARE operates in one or more defined membrane locations, which can differ depending on cell type. They form the minimal machinery required for membrane fusion and loss of any one component of a specific *trans*-SNARE complex inhibits fusion at distinct sites in the cells (29, 30). For example, deletion or mutation of VAMP3, a recycling endosome SNARE, will inhibit secretion of pathways that require this SNARE for delivery of recycling endosome membrane to the cell surface (11, 31, 32). Thus, SNAREs have been employed to map a number of cytokine secretion pathways in macrophages and many other cell types.

**Figure 3:**
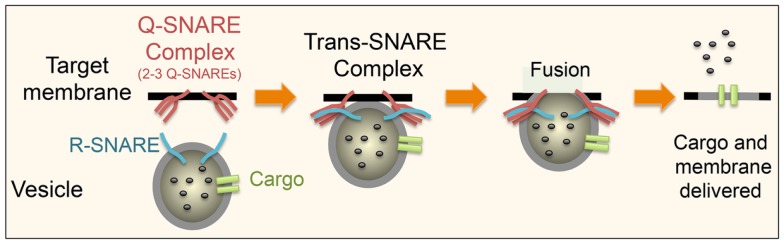
**Schematic showing the basic steps in SNARE-mediated secretion is shown**. An R-SNARE on the donor membrane comes together with a Q-SNARE complex, consisting of two to three Q-SNAREs, on the target membrane to form a trans-SNARE complex. This brings the two membranes into close proximity leading to the formation of a fusion pore allowing the release of some or all of the contents of the vesicle. The pore can either close (“kiss and run”) or as depicted above the membrane can fully fuse thus incorporating all of its membrane-associated proteins into the target membrane. This basic process is same in all cell types and at different stages of the transport pathways. What differs is the precise SNARE partners at these distinct stages.

SNARE proteins are mostly transmembrane proteins, with only a few of them existing as peripheral membrane proteins, anchored to membrane lipids by post-translational modifications and/or through protein–protein interactions. Membrane anchored heterologous SNAREs from opposing membranes form a tight four-helix bundle, where each helix is contributed by one of four SNARE motifs, which act to pull the two membranes into close proximity (30, 33) (Figure 3). The energy released when the membrane-bridging (trans-) SNARE complex forms initiates the formation of a fusion pore between the two opposing membranes. Cargo (or a portion of it) is discharge through this opening, which might subsequently close in a process known as “kiss and run exocytosis,” or alternatively, the whole vesicle can fully fuse, merging transmembrane proteins and lipids into the target membrane and fully releasing its lumenal contents (34, 35). In the latter case, membrane and resident proteins – importantly including incoming SNAREs – are then retrieved by endocytosis, which in the case of macrophages is especially important as large quantities of membrane are turned over at cell surface during processes such as macropinocytosis and phagocytosis.

SNARE subtypes have been described based on their central amino acid, a glutamine (Q-SNARE) or an arginine (R-SNARE). Based on the position of their SNARE motif in the trans-SNARE four helix bundle, Q-SNAREs are further partitioned into one of four subgroups: Qa, Qb, or Qc or, in the case of SNAREs like SNAP23 that contribute two SNARE motifs, Qb,c (36, 37). Thus, a trans-SNARE complex consists of one R-SNARE and Q-SNARE complex, comprising two to three Q-SNAREs (Qa, Qb, and Qc or Qa and Qb,c), which are typically found on opposing membranes. The specificity of SNARE pairing to form individual complexes is in part dictated by the locations of family members. After fusion, the SNARE complex then resides in the donor membrane and its subunits must be rapidly disassembled by the ATPase NSF with its cofactor SNAP, and recycled to their original sites for further use (38).

Over 60 mammalian members of the small GTPase family of Rabs help to regulate trafficking at multiple steps through trafficking pathways [reviewed in Ref. (39, 40)]. Like other GTPases, Rabs switch between an inactive GDP-bound form (in the cytoplasm) and an active GTP-bound form (on membranes). The Rab cycle and nucleotide switching is aided by a host of accessory proteins. Rabs have varied roles in trafficking through effectors that can include phosphoinositide-modifying enzymes, budding machinery, molecular motors, tethering factors, and SNAREs (41–43). Through tethering effectors, Rabs orchestrate the docking and tethering of vesicles prior to SNARE-mediated fusion on sequential organelles in transport pathways. Experimentally, Rabs can be assigned to transport steps through the actions of GDP- and GTP-bound functional mutants, through deletion or genetic mutation of Rab genes, including those associated with human disease. Macrophages have a rich array of Rabs and many of their immune functions include regulation by Rab cascades. Notably also, key Rabs are targeted by bacterial effectors of intracellular pathogens.

Molecular regulators, like the Rabs and SNAREs that are found throughout the different trafficking pathways, offer many potential targets for therapeutic intervention, although inflammation has not yet directly benefited from these approaches (44). SNAREs are notably inactivated by botulinum and tetanus toxins and the therapeutic potential for this approach in wide ranging conditions is actively pursued (45). Rab proteins are also considered as attractive therapeutic targets, including via prenylation inhibitors (46). Future studies will no doubt increasingly explore some of these solutions for controlling cytokine secretion.

## TNF Secretion

### Sorting and transport of TNF

TNF mRNA is constitutively expressed and controlled by mRNA stability and translation. Activation leads to its rapid transcription, generating a type II membrane protein that can be found in the macrophage Golgi complex soon after LPS-activation (47). TNF is then packaged exclusively into a population of TGN-derived tubules/vesicles labeled with the GRIP-golgin p230/golgin245 (14). The GRIP-domain golgins are located on distinct TGN domains and regulate trafficking to and from the TGN. Four mammalian GRIP-golgins exist on the TGN, namely p230, CCC88, GCC185, and golin-97, and all share a TGN targeting GRIP domain in their c-terminus (48). What is notable is that in the absence of LPS, similar numbers of p230 and golgin-97 labeled tubules/carriers exit the TGN but upon activation with LPS the p230-labeled tubules are selectively upregulated threefold to enhance cytokine transport (14).

The p230-labaled TNF carriers are also regulated by Rab6 and Rab6a′, which act to stabilize Arl1-recruited p230 on TGN membranes (15). The Rab6 isoforms are quintessential Golgi-associated Rabs and both participate in multiple steps of trafficking through several effectors. Myosin IIA is a Rab6 effector on the p230-labeled tubules that move TNF and other cargo from the TGN to recycling endosomes (49). Rab6, like other Rabs involved in the macrophage constitutive pathway, is upregulated by LPS, offering more transport capacity in activated cells.

Lipids and phospholipids at the TGN are also regulated for trafficking. Despite the preponderance of PI4P in Golgi membranes, a number of PI3 kinases regulate budding and vesicle exit from the TGN (50). Among them, PI3Kδ is found on TGN membranes, where it and dynamin II, regulate TNF trafficking and TGN exit (23). The GTPase dynamin II functions in fission of clathrin-coated and non-clathrin-coated vesicles and carriers at the Golgi and on other membranes (51). A number of lipid metabolic genes are also upregulated in response to LPS, including choline cytidylyltransferase alpha (CCTα), phospholipase D1 (PLD1), and the choline/ethanolamine phosphotransferase C/EPT (20). Lipids play an active role in the biogenesis of carriers and in organizing TGN exit domains (20). Thus, localized changes in lipid composition can greatly alter secretion by recruiting specific proteins and promoting membrane fission or fusion. CCTα, a key enzyme catalyzing PtdChol biosynthesis in macrophages, regulates vesicle formation and budding (20). Its inactivation reduces PtdChol synthesis leading to decreased TNF secretion suggesting that PtdChol and CCTα are also critical for transport from the Golgi complex (20). Thus, the upregulation of lipid machinery also enhances the export of TNF from the TGN.

TGN carriers transport TNF and other cargo to recycling endosomes as a second transit and sorting station. This transport is well-defined through identification of the relevant SNAREs (11, 12). The Q-SNARE complex Stx6/Stx7/Vti1b is packaged with TNF into TGN-derived vesicles and upon reaching the recycling endosome, this Q-SNARE complex pairs with the resident R-SNARE VAMP3 for fusion (11, 12). The members of this SNARE complex are upregulated and packaged into the TGN-derived vesicles in response to LPS to accommodate the increased number of p230 requiring fusion with the recycling endosome (11, 12). On-going transport to the cell surface requires the recycling endosomes, or tubular extensions of this compartment bearing VAMP3, to fuse with the plasma membrane, via the cell surface Q-SNARE complex of Stx4/SNAP23 (11, 22). This step of the constitutive pathway, like earlier stages, is also enhanced in activated macrophages by upregulation and increased levels of the cell surface SNAREs (11, 22). Loss or inactivation of any of these SNARE components blocks TNF secretion.

The location of the surface SNARE complex plays a key role where TNF secreted. Surface SNAREs, such as Stx4 are clustered in cholesterol-rich lipid rafts, which adorn filipodia and phagocytic cups on the surface of activated macrophages (52). Disruption of these lipid rafts reduces TNF secretion (52). Macrophages use their filopodia to explore their environment and capture pathogens; this binding leads to filopodia retraction and pseudopod formation from the underlying lamellipodia to form the phagocytic cup (53). Stx4 translocates and concentrates on these lipid rich pseudopods and regulates the focal fusion of the recycling endosome with the phagocytic cup (11, 52). This serves two purposes; providing extra surface membrane necessary for macrophages to engulf microbes while simultaneously delivering TNF to the pseudopod tips for rapid secretion (11, 52). Stx4 is then removed, and presumably recycled to the cell surface, after the phagosome is internalized (11, 52).

Two Rab GTPases, Rab11 and Rab37, have been found to regulate surface delivery of TNF (11, 16) and these Rabs are also upregulated after LPS activation of cells. Rab11 is a well-known marker of recycling endosomes, where it mediates transport to the cell surface and similarly participates in the delivery of TNF through recycling endosomes to the plasma membrane. Rab37 is also located on the TNF-loaded vesicles that fuse with the plasma membrane. Intriguingly, in macrophages Rab37 associates with Munc13-1, a diacylglycerol, calcium, and calmodulin activated SM family protein, known to be required for synaptic vesicle priming and for insulin release in pancreatic beta cells (16). Loss of Munc13-1 or Rab37 reduced TNF secretion (16).

Adaptor proteins regulate sorting of membrane proteins and the AP-I complex has been implicated in both phagocytosis and the trafficking of TNF from the recycling endosome (24). Typically, AP complexes recruit the coat protein clathrin to vesicles for their formation, but this is not the case during phagocytosis where AP-1 decorated endosomes amass below the phagocytic cup in an ARF-1-dependent, clathrin-independent manner (24). Depletion experiments suggest that AP-1 might act to sort TNF at the recycling endosome and a cleaved form of AP-1 accompanies carriers that bud off the recycling endosome to the surface for VAMP3- and ARF6-dependent delivery at the tips of pseudopodia during phagocytosis (54, 55).

The protein trafficking machinery described above largely regulates discrete stages of TNF secretion, but this may not be the case for all trafficking machinery. Secretory carrier membrane proteins (SCAMPs) are found in complexes with SNARE proteins and regulate exocytosis (21). In macrophages, SCAMP5 expression is upregulated by Toll-like receptors (TLR) agonists like LPS and by increases in intracellular calcium through stimulation with ionomycin (21). Both stimuli result in SCAMP5-dependent TNF secretion (21). SCAMP5 has the potential to regulate SNAREs at multiple points in the TNF secretory pathway and can form complexes with many of the SNAREs (Stx4, Stx6, SNAP23, and VAMP3) identified at the different stages of TNF secretion (21). In the absence of stimulus, SCAMP5 localizes predominantly to the Golgi complex along with Stx6 and after stimulation with ionomycin shifts to the recycling endosome and cell surface, where it colocates with Stx4/SNAP23 (21). This shift in localization occurs in parallel to TNF secretion (21). Although SCAMP5 forms a complex with SNARE proteins, this binding is not direct and occurs through the calcium binding transmembrane synaptotagmins (Syt) (21). Loss of the synaptotagmin-binding site in SCAMP5 inhibits ionomycin induced TNF secretion (21). SCAMP5 binds at least two synaptotagmins, Syt1 and Syt II, but their role in TNF has yet to be established.

However, other synpatotagmins play a role, or have been implicated in, TNF secretion. Syt V is located on recycling endosomes and filopodia-like structures and is recruited to the nascent phagosome (56). It has a role in the recruitment of recycling endosomes to the phagocytic cup, and its loss impairs phagocytosis suggesting that it might also positively regulate TNF secretion (56).

While Syt V’s role in TNF secretion has not been tested directly, another synaptotagmin, Syt XI, located on recycling endosomes and recruited to phagosomes, has been found to inhibit TNF secretion. Its role in inhibiting cytokine secretion suggests that members of this family of proteins might play an important role in regulating different aspects of cytokine secretion (25). Syt XI is one of the few family members that does not bind calcium and unlike most family members, it inhibits vesicle fusion by an unknown mechanism but one that could be focused on inhibiting SNARE function. Syt XI also inhibits IL-6 secretion, whose trafficking diverges from TNF at the level of the recycling endosome, suggesting that Syt XI might function prior to surface delivery. Taken together, protein families, like SCAMPs and synaptotagmins, functioning alongside SNAREs, also clearly participate in cytokine release at or near surface domains.

Rho GTPases act as molecular switches, that cycle between GTP-bound inactive and GDP-bound inactive states, to regulate remodeling of the actin cytoskeleton in cellular processes such as adhesion, migration, and membrane trafficking. Rac1 and CDC42 regulate the cell surface leading to the formation of lamellipodia and smaller filapodia, respectively, and also play a role in regulating organelle movement (57, 58). In LPS stimulated macrophages, these two Rho GTPases are essential for efficient surface delivery of TNF (26). In the absence of Rac1 function in particular, TNF is synthesized and is held up in recycling endosomes along with other endosomal cargo such as transferrin (26). These results suggest that Rac1 is involved in the final stages of TNF transport to the cell surface, possibly through the positioning or surface delivery of TNF-loaded recycling endosomes.

### TACE-mediated release of soluble TNF

Once incorporated into the cell surface, membrane anchored TNF is cleaved by the ADAM family metalloproteinase TNF-converting enzyme (ADAM17 or TACE) at a site after alanine 76, to release the soluble active TNF ectodomain. Both transmembrane proteins, TACE and TNF (proprotein) can be organized by lipid raft domains on the cell surface (52). In different cell types and contexts, TACE can be found sequestered in lipid rafts or in non-raft fractions. In macrophages, TACE appears primarily in non-raft fractions, whereas TNF is delivered initially to lipid rafts in the plasma membrane where the Stx4 Q-SNARE complex is enriched for membrane fusion (52, 59). TNF is delivered to nascent phagocytic cups, where TACE is also enriched (11, 52). One possible level of regulation for cytokine release is the movement of TNF out of lipid rafts to access TACE for cleavage.

The level of TACE at the cell surface is also modulated by LPS cell activation and by intracellular trafficking machinery (19). Recently, the intramembrane protease, iRhom2, has been identified as a key regulator of TACE. Although catalytically inactive, iRhom2 is a member of the rhomboid family of intramembrane serine proteases. At the ER, TACE is bound by iRhom2, which promotes its exit from the ER and loss of iRhom2 leads to a build up of TACE in the ER (19). Both the pro and mature forms of TACE bind to iRhom2 suggesting that it remains associated through multiple stages of the secretory pathway (60). Loss or inactivating mutations of iRhom2 in mice reduce the levels of TNF secreted in response to LPS challenge (61). These results together suggest that iRhom2 acts as a cargo receptor or chaperone to aid in the trafficking of TACE from the ER to the Golgi where furin activates TACE by cleaving its N-terminal inhibitory domain (19). Like many proteins that regulate TNF secretion, LPS stimulation upregulates iRhom2 levels, permitting increased TACE transport to the cell surface for TNF cleavage and shedding (19).

## Interleukin 6 Secretion

Interleukin 6 (IL-6) is another proinflammatory cytokine and an example of soluble cargo transported through the constitutive secretory pathway. At the level of the TGN, some IL-6 can be segregated away from TNF into distinct carriers (10). The rest appears together with TNF in post-Golgi carriers and in recycling endosomes (10). Like TNF, IL-6 transport out of the TGN relies on PtdChol and CCTα (20). The R-SNARE VAMP3 on the recycling endosome regulates the delivery of Q-SNARE Stx6/Vti1b/Stx7 labeled vesicles containing IL-6 (10).

At the recycling, endosome IL-6 and TNF are segregated into distinct domains and transported to the plasma membrane through different routes (10). Exactly how they are sorted in the recycling endosome is unknown, but it maybe that TNF is actively sorted away from IL-6 or alternatively both are individually sorted. Not surprisingly, a number of trafficking machinery proteins that regulate TNF at the level of the recycling endosome, including SCAMP5 and Syt XI also regulate IL-6 secretion (21, 25). While both TNF and IL-6 are delivered to the cell surface, the transport route for IL-6 from the recycling endosome is multidirectional even during phagocytosis when TNF is specifically delivered to the tips of nascent phagosomes for secretion (10).

## Interleukin-10 Secretion

Interleukin-10 (IL-10) is also a soluble cytokine targeted to the lumen of the ER by a signal peptide. In RAW cells, IL-10 secretion begins later than that of TNF and IL-6 and persists for longer (13). Importantly, IL-10 is a regulatory cytokine and one of its primary effects is to decrease the secretion of TNF and other proinflammatory cytokines. From the ER, newly synthesized IL-10 is transported to the Golgi complex where at the TGN, it can be directed into at least two distinct pathways to the surface, one direct and the other indirect. The latter pathway carries the bulk (80%) of IL-10 in the same p230 labeled carriers as IL-6 and TNF to the recycling endosome, where it is sorted for transport to the cell surface (13). Depletion or inactivation of p230/golgin245 or other TGN machinery disrupts IL-10 secretion through this recycling endosome route (13) and the final surface delivery of IL-10 is enacted by Rab11 and VAMP3-positive recycling endosomes, which fuse with the plasma membrane (13). Thus, there is no separation of pro- and anti-inflammatory cytokines in terms of pathways utilized.

In the second secretory pathway, IL-10 is packaged with ApoE at the TGN into golgin-97 labeled tubules and transported directly to the cell surface, bypassing the recycling endosome (13). This ApoE transport pathway is dependent on microtubules and PKA (13). As a soluble cytokine, IL-10 is likely to be packaged into multiple routes for TGN exit and surface delivery. Redundancy in overall pathways, carriers, and molecular regulators for the secretion of different cytokines, such as IL-6 and IL-10, reflects the fact that many are soluble proteins, and therefore, handled as “bulk cargo” by the cell. As a deeper understanding of trafficking emerges, especially in post-Golgi steps, factors such as sorting receptors, membrane domains, and intraluminal pH, may well be found to differentiate the final release of soluble cytokines like IL-6 and IL-10.

## Interleukin-1 Beta Secretion

Members of the IL-1 cytokine family, particularly IL-1β, IL-18, and IL-37 are key inflammatory cytokines with important roles also in disease, and with the distinction of using non-classical or unconventional secretory pathways for their release (62). In macrophages activated through a TLR, the precursor pro-IL-1β is synthesized but to fully achieve cytokine release, a second danger signal, for instance extracellular ATP (63), activates the multisubunit signaling complex of the NLRP3/caspase-1 inflammasome. Activated caspase-1 proteolytically cleaves pro-IL-1β to produce the mature, active form of the cytokine that is secreted. Exactly how IL-1β and other such cytokines find their way out of cells remains a topic of conjecture and conflicting evidence (62).

Several routes have been championed for the release of cleaved IL-1β from monocytes/macrophages and in different circumstances any or all of these routes may apply. Earlier studies on human monocytes used inhibitors to implicate the ATP binding cassette transporter (ABC1) as an exit portal directly through the plasma membrane for the release of IL-1β (64). The ABC transporter route has also been demonstrated for other soluble, secreted mediators including macrophage migration inhibitor factor (MIF) (65). Cell death through pyroptosis and other mechanisms may also lead to leakage and release of leaderless cytokines (66). Unconventional vesicular or organellar pathways have also been touted. Exosomes, derived either by blebbing of the plasma membrane or by budding off endosomal/lysosomal compartments may enfold cytosolic IL-1β for transport out of the cell [reviewed in Ref. (67)]. Macrophages have secretory lysosomes (68), which equate in some respects to LROs as dedicated secretory compartments in some other cell types. There is evidence for the transfer (by an unknown transporter) of leaderless cytoplasmic proteins, including cytokines (69), into secretory lysosomes for release. Related organelles, autophagosomes, or autophagolysosmes, have also been implicated in IL-1β release. While autophagy is primarily a degradative process in the cytoplasm, secretory autophagy is proposed as a parallel function for unconventional secretion, in yeast (70) and mammalian cells (71). This secretory autophagy may contribute transiently to acute release of IL-1β in activated macrophages, via a pathway that interestingly is mediated by Atg5, inflammasome, the Golgi reassembly stacking protein 55 (GRASP55) and Rab8a (72). Knockdown of Rab8a reduced the secretion but not the synthesis of IL-1β (72); moreover, Rab8a and its close homolog Rab8b have distinct roles in secretory and degradative autophagy, respectively (71). Further studies are likely to reveal more of the machinery associated with these unconventional secretory routes, predictably more Rabs, and also possibly SNAREs for vesicular routes.

## Other Cytokines

To date, little data are available on the trafficking machinery necessary for the secretion of many of the cytokines secreted by macrophages. For some, it has been shown specific subunits or receptors can chaperone cytokines through the secretory pathway. IL-15 and IL-12 are two such cytokines. IL-12 is a proinflammtory cytokine that controls the differentiation of naïve T cells into IFN-γ-producing Th1 cells and plays key a role in the development of autoimmune diseases and in the pathology associated with inflammatory related disease such as psoriasis (73). The bioactive form of IL-12, known as p70, is composed of two subunits, P35 and p40, with the availability of p35 being the major determining factor in its secretion (74). P35 is regulated at both transcriptional and post-transcriptional levels, and like p40 contains a signal peptide that targets it to the ER (75). However, in contrast to p40 whose signal peptide is removed during translocation into the ER, the p35 signal peptide is cleaved in an unconventional manner (75). Within the ER, a primary intermediate cleavage occurs, followed by a second cleavage before or immediately after exit from ER (75). Once in the ER p35, an integral membrane protein, binds to soluble p40 subunit to form membrane anchored p70 heterodimer, which then leads to its transport through the Golgi complex to the cell surface (76).

IL-15 plays an important role in regulating the activity and persistence of a large variety of cell types. Much of the work on IL-15 secretion has been undertaken in other cell types where its secretion, via the ER and Golgi complex, is dependent on its cognate receptor, the IL-15 receptor α (IL-15Rα), which acts as a chaperone enabling IL-15 to exit the ER and traffic through the secretory pathway (77). In macrophages, IL-15Rα is upregulated by IFNγ treatment, leading to secretion of IL-15, suggesting that a similar chaperone mechanism might occur (77). IL-15Rα contains a nuclear export sequence and can also be transported to the cell surface via the nucleus along with IL-15 in a chromosomal region maintenance 1 (CRM1) dependent manner (78). ARF6 has also been shown to promote IL-15 secretion, although exactly how is not clear (78).

The DNA binding nuclear protein high-mobility group box 1 protein (HMGB1) has multiple roles including being a potent mediator of inflammation (79). Extracellular HMGB1 acts as a proinflammatory alarmin or DAMP (damage-associated molecular pattern) that regulates inflammation through its binding to TLR 2, TLR4, and TLR9, to CD42 and to the advanced gylcation end product receptor (RAGE) (79). It can be released passively during necrosis (80) or actively through at least one non-classical secretory pathway (72, 81). Secretion begins around 6 h post-stimulation with levels still increasing 30 h later suggesting that it has a “late” role in regulating inflammation (81). Lacking a secretory signal peptide, HMGB1 is not targeted to the ER and Golgi complex. It does possesses a nuclear localization signal (NLS) and two non-classical nuclear export signals (NES) that regulate its shuttling between the cytosol and nucleus, with its equilibrium almost completely shifted toward nuclear accumulation in unactivated monocytes and macrophages (82). On activation, phosphorylation and acetylation of HMGB1 target it to the cytosol and partially into lysosomes from where it can be secreted (81, 82). The trafficking machinery that regulate this pathway and its release from cells are unknown, although in cancer cells HMGB1 is found in a complex with Annexin A2, myosin IC isoform a, myosin 9, and Rab10, suggesting that these proteins might play a role in its release (83). In macrophages, the same autophagy-based unconventional secretion pathway that releases IL-1β can also secrete HMGB1 and accordingly its release has the potential to be regulated by Atg5, the inflammasome, the Golgi reassembly stacking protein 55 (GRASP55), and Rab8a (72). Thus HMGB1 might be released by two different routes, although whether this occurs simultaneously or under distinct circumstances is yet to be determined.

## Conclusion

The sequential and temporally orchestrated nature of cytokine secretion limits the demand for increased secretion to distinct times of need. It is becoming increasingly clear that upon LPS-activation macrophages adapt to this mandate by resourcefully modifying the levels or location of specific trafficking machinery molecules. This bolsters the sorting of cargo at the two major sorting hubs – the TGN and recycling endosome – and accelerates the passage of cytokines through distinct transport pathways. Although cytokine trafficking has not directly been compared in macrophages under different activation regimes, the overlapping pathways used to traffic IL-10 compared to IL-6 for instance, would suggest that both M2 and M1 polarized cells have largely common secretory pathways.

The adaptations for some of the cytokine trafficking pathways are strategic, such as the routing of recycling endosome membrane containing TNF to the phagocytic cup during times of infection. This clever strategy allows the concomitant release of TNF and provides extra membrane for phagocytic cup formation processes. Other adaptations might allow the packaging of cytokines into more than one pathway for rapid release. Whether non-classical secretory pathways are similarly modified during infection is unknown, but answering this along with further elucidation of the classical pathways and machinery might in future allow the targeted intervention in inflammatory diseases where excessive cytokine secretion is a major determining factor.

## Conflict of Interest Statement

The Guest Associate Editor Paige Lacy declares that, despite having collaborated with author Jennifer Stow, the review process was handled objectively and no conflict of interest exists. The authors declare that the research was conducted in the absence of any commercial or financial relationships that could be construed as a potential conflict of interest.
